# Ferroelectric Polarization-Enhanced Photocatalysis in BaTiO_3_-TiO_2_ Core-Shell Heterostructures

**DOI:** 10.3390/nano9081116

**Published:** 2019-08-03

**Authors:** Xiaoyan Liu, Siyi Lv, Baoyan Fan, An Xing, Bi Jia

**Affiliations:** 1 Chongqing Key Laboratory of Nano/Micro Composites and Devices, College of Metallurgy and Materials Engineering, Chongqing University of Science and Technology, Chongqing 401331, China; 2 Shenzhen Key Laboratory of Nanobiomechanics, Shenzhen Institutes of Advanced Technology, China Academy of Sciences, Shenzhen 518055, China

**Keywords:** ferroelectric polarization, internal electric field, charge separation and transport, heterostructured photocatalysts

## Abstract

Suppressing charge recombination and improving carrier transport are key challenges for the enhancement of photocatalytic activity of heterostructured photocatalysts. Here, we report a ferroelectric polarization-enhanced photocatalysis on the basis of BaTiO_3_-TiO_2_ core-shell heterostructures synthesized via a hydrothermal process. With an optimal weight ratio of BaTiO_3_ to TiO_2_, the heterostructures exhibited the maximum photocatalytic performance of 1.8 times higher than pure TiO_2_ nanoparticles_._ The enhanced photocatalytic activity is attributed to the promotion of charge separation and transport based on the internal electric field originating from the spontaneous polarization of ferroelectric BaTiO_3._ High stability of polarization-enhanced photocatalysis is also confirmed from the BaTiO_3_-TiO_2_ core-shell heterostructures. This study provides evidence that ferroelectric polarization holds great promise for improving the performance of heterostructured photocatalysts.

## 1. Introduction

Organic synthetic dyes in wastewater has become a hard-to-solve source of pollution, causing serious environmental and health problems because of their high solubility and chemical stability in water. Several techniques, including physical adsorption and biological or chemical treatments, have been developed for dye degradation, but they are limited in terms of their practical applications due to their intrinsic disadvantages caused by harmful active byproducts [1,2]. Photocatalysis is a promising pathway for wastewater purification, in which photogenerated charges in catalysts are reacted with water to induce actives of hydroxyl radicals (OH^•^) and superoxide anions (^•^O_2_^−^) for dye degradation [3]. To achieve an effective degradation, catalysts need to possess the following characteristics: broad light absorption, efficient charge separation and transport, as well as good chemical stability. A large amount of photocatalysts including oxide semiconductors [4,5] and sulfide semiconductors [6,7] with a comparatively high degradation efficiency have been reported. Among them, TiO_2_ is one of the most widely used photocatalysts, benefiting from its excellent chemical stability, non-toxicity, and low cost. Mesoporous TiO_2_ nanoparticles have a large surface area, which is favorable for the creation of numerous active sites for photocatalytic reactions [8,9]. However, the mesoporous TiO_2_ cannot fully support space charge effects for the separation of photogenerated charges as a result of its nanoscale grain size [10]. Because the photogenerated charges and photocatalytic reaction products are produced in close proximity to each other, the relatively high rate of charge recombination in mesoporous TiO_2_ nanoparticles often results in a low quantum yield and poor efficiency of photocatalytsis [11]. Accordingly, it is necessary to develop functional photocatalysts with suppressed charge recombination.

One potential solution is to build an internal electric field in heterostructured photocatalysts. An internal electric field in heterostructured photocatalysts can arise from ferroelectric polarization, polar surfaces, p-n junctions, and polymorph junctions [12]. In ferroelectrics, spontaneous polarization due to displacements of atoms or ions in the crystal lattice creates high-density charges on the surfaces of both ends of the polarization vector [13,14]. When a ferroelectric material is involved in a heterostructure, polarization charges in ferroelectrics cannot be fully compensated by low-concentration free charges in the adjacent semiconductor_,_ resulting in an internal electric field in the heterostructure. Several studies have demonstrated the effectiveness of implementing ferroelectric polarization to improve the performance of semiconductor devices through a polarization-induced internal field. For instance, ferroelectric LiNbO_3_ and BaTiO_3_ were employed to facilitate charge separation and transport in dye-sensitized solar cells to increase cell efficiency [15,16]. Recently, applications of ferroelectric polarization were also explored to enhance photocatalytic performance [17,18,19]. Although ferroelectric-enhanced photocatalysis has been verified by various experiments, the mechanism remains unclear. In addition, the wide band gap of typical ferroelectric materials exclude their single phases from the applications depending on light absorption efficiency. In this paper, we report an approach of ferroelectric polarization-enhanced photocatalysis on the basis of BaTiO_3_-TiO_2_ core-shell heterostructures synthesized via a hydrothermal process. Through an optimized hydrolysis reaction, mesoporous TiO_2_ nanoshells were coated on BaTiO_3._ In comparison with pure TiO_2_ nanoparticles, a higher photocatalytic performance was seen in the BaTiO_3_-TiO_2_ core-shell heterostructures of all weight ratios adopted in this work, which was due to the lower recombination rate of photogenerated electron-hole pairs as evident from photoluminescence (PL) spectroscopy [20,21,22,23]. The internal field arising from ferroelectric BaTiO_3_ core acts as a driving force for the separation of photogenerated electron-hole pairs and promotion of charge transport to the surface of TiO_2,_ resulting in increased photocatalytic activity with excellent stability. This approach provides a successful demonstration of the ferroelectric polarization-induced internal field in heterostructured photocatalysts as an effective strategy for improving the photocatalytic performance.

## 2. Materials and Methods

### 2.1. Material Preparation

A hydrothermal process [24] was employed to synthesis the BaTiO_3_-TiO_2_ core-shell heterostructures, and the meroporous TiO_2_ nanoparticles as a reference sample. Commercial BaTiO_3_ crystallines (99%, KJ Group, Hefei, China) were ultrasonically dispersed in ethanol, followed by the addition of hexadecylamine (HDA, 90%, Sigma-Aldrich, St. Louis, MO, USA) and NH_3_·H_2_O, and the mixture was ultrasonicated continuously at room temperature for 10 min to create a homogeneous solution. Subsequently, titanium isopropoxide (TIP, 95%, Sigma-Aldrich, St. Louis, MO, USA) as the precursor of TiO_2_ was dropwise added into the above solution under stirring for a hydrolysis reaction of 12 min. NH_3_·H_2_O is used as a catalyst to promote the hydrolysis reaction of TIP. HDA is used as a surfactant segregated to the surface of BaTiO_3_ crystallines. Hydrogen-bonding interactions between HDA molecules and TIP oligomers occur to generate inorganic–organic composites that coat BaTiO_3_. The amount of TIP (0.12, 0.16, 0.2 mL) and NH_3_·H_2_O (0, 0.1, 0.2 mL), and the stirring rate during the hydrolysis reaction (160, 200, 240 rpm) were adjusted to build core-shell geometries.

The resultants of BaTiO_3_-TiO_2_/HDA nanocomposites were collected by centrifugation. After washing with ethanol and deionized (DI) water, the BaTiO_3_-TiO_2_/HDA nanocomposites were dispersed in a mixture of 20 mL ethanol and 10 mL DI water, and then sealed in a teflon-lined autoclave. The hydrothermal reaction was proceeded at 160 °C for 16 h. The HDA molecules could be removed with the solvothemal treatment at 160 °C to generate mesoporous in TiO_2_ that remained as the nanoshells. The obtained BaTiO_3_-TiO_2_ samples were washed with ethanol and DI water to remove residual organics, then dried at 80 °C for 12 h.

BaTiO_3_-TiO_2_ core-shell heterostructures with different weight ratios of BaTiO_3_ to TiO_2_ (1:1, 1.2:1, 1.4:1) were synthesized through the hydrolysis reaction followed by the hydrothermal treatment. The optimum synthesis condition was verified by checking photodegradation efficiency of the catalysts synthesized at the different amount of TIP and NH_3_·H_2_O, and the varied stirring rate during the hydrolysis reaction, as shown in Figure S3. Meanwhile, mesoporous TiO_2_ nanoparticles were synthesized at the absence of BaTiO_3_ while the other conditions remained unchanged.

### 2.2. Characterization

X-ray diffraction (XRD, DX-2700 diffractometer) with a Cu Kα radiation (35 kV, 25 mA) was used to record phase structures of the as-synthesized BaTiO_3_-TiO_2_ core-shell heterostructures and TiO_2_ nanoparticles, and the commercial BaTiO_3_ crystallines. The morphology and crystal structure of the samples were characterized by a high resolution transmission electron microscope (HRTEM, JEM-2100F, JEOL Ltd., Tokyo, Japan and a field-emission scanning electron microscopy (FESEM, JSM-7800F, JEOL Ltd., Tokyo, Japan). Brunauer–Emmett–Teller (BET) surface areas of the samples were analyzed by Quadrasorb 2MP using N_2_ as the adsorption gas. Ferroelectricity of the BaTiO_3_ crystallines following the hydrothermal process was validated using an atomic force microscopy (AFM, Cypher™, Oxford Instruments, Goleta, CA, USA) via recording phase-voltage hysteresis and amplitude-voltage butterfly loops with switching spectroscopy piezoresponse force microscopy (SSPFM) [25], which is an effective tool for demonstrating of local ferroelectric characteristics. The photoluminescence (PL) spectra were recorded using PL mapping system (iHR320, Horiba Ltd., Kyoto, Japan) at an excitation wavelength of 325 nm ranging 300–600 nm.

### 2.3. Photocatalytic Activity Measurements

The photocatalytic activity was evaluated by degradation of rhodamine-B dye (RhB) solution under UV light irradiation. Photocatalysts of 30 mg were mixed into 50 mL of 15 ppm RhB solution with pH value of 5.7 ± 0.5. A UV spotlight (L9566-02, Hamamatsu Photonics, Hamamatsu, Japan) equipped with a 200 W mercury-xenon lamp and a 280–400 nm filter was used as the UV light source. The total optical power impinging on the mixture solution was 10 mW/mL. Prior to UV light irradiation, the mixture was stirred in the dark for 30 min to reach desorption–absorption equilibrium. A suspension of 3 mL was drawn out at the given time and then centrifuged at 8000 rpm to remove the photocatalyst completely. The RhB degradation rates were calculated from the UV-vis absorption spectra of the supernatant measured by a UV-vis spectrophotometer (Persee T10, PERSEE Analytics, Auburn, CA, USA) over the range 300–700 nm. The calibration curve was run at the maximum peak of 554 nm, as seen in Figure S5.

## 3. Results and Discussion

BaTiO_3_ is a typical perovskite ferroelectric with a Curie temperature around 120 °C. Below the Curie temperature, phase transition from a cubic paraelectric to a tetragonal ferroelectric occurs accompanied with lattice distortion due to Ti ions shift along the [001] axis, as seen in Figure S1, producing a spontaneous polarization (*P*_s_ = 27 µC/cm^2^) [26]. In this work, commercial BaTiO_3_ crystallines were used as the core material. After being subjected to the hydrothermal process, the BaTiO_3_ crystallines with a size of 100–300 nm showed excellent ferroelectricity, as seen in Figure S2, which coincides with the size-dependent ferroelectric property in BaTiO_3_ [27].

**Figure 1:**
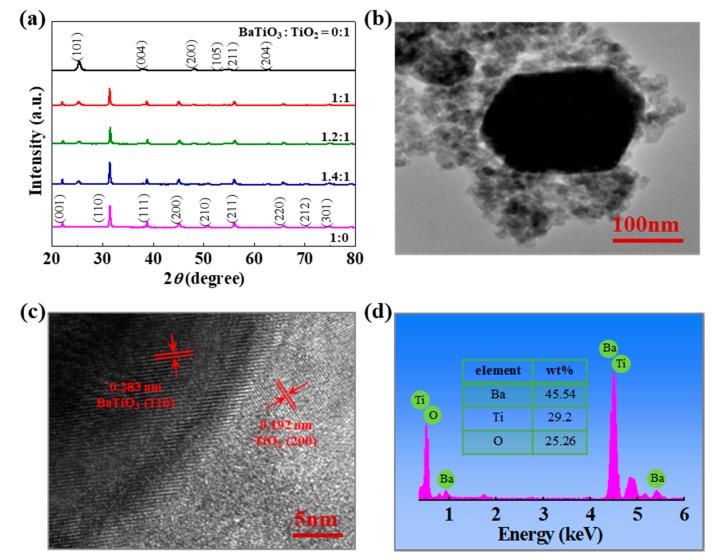
(**a**) X-ray diffraction (XRD) patterns of the as-synthesized BaTiO_3_-TiO_2_ composites and TiO_2_ nanoparticles, and commercial BaTiO_3_ crystallines after being subjected to the hydrothermal process. high resolution transmission electron microscope (HRFEM)images at low solution (**b**) and high solution (**c**) of the BaTiO_3_-TiO_2_ composite (1.2:1), (**d**) energy dispersive X-ray spectrometry (EDS) analysis of the BaTiO_3_-TiO_2_ composite.

As seen in Figure 1a, the XRD patterns confirm that all the BaTiO_3_-TiO_2_ composites consist of tetragonal BaTiO_3_ (JCPDS 05-0626) and anatase TiO_2_ (JCPDS 21-1272). All the diffraction peaks can be indexed as well-crystallized BaTiO_3_ and TiO_2_. No impurity peaks were found in the BaTiO_3_-TiO_2_ composites, implying no impurity phases formed in the composites. The representative structure of an individual BaTiO_3_-TiO_2_ composite (1.2:1) was characterized by a field emission TEM (FETEM). The low-resolution FETEM image shows a distinguishable core-shell structure that the outer shell consists of mesoporous TiO_2_ nanoparticles at a thickness less than 100 nm, as seen in Figure 1b, which is comparable to the depletion width [28]. A high-magnification FETEM image revealed a good lattice match of the core-shell structure. The lattice spaces of about 0.283 nm and 0.192 nm measured from the core and shell portions are in an agreement with the (110) and (200) planes of tetragonal BaTiO_3_ and anatase TiO_2,_ respectively, as seen in Figure 1c. The BaTiO_3_-TiO_2_ core-shell configuration was further confirmed by the energy dispersive X-ray spectrometry (EDS) analysis, as seen in Figure 1d, and line-scan EDS profile crossing the core-shell structure, as seen in Figure S4b.

Surface area of photocatalyst is an important factor influencing photocatalysis; a larger surface area can provide more active sites on the surface for photodegradation of organic dyes. BET surface areas of all the samples used in this work were examined and are listed in Table 1. The BaTiO_3_-TiO_2_ core-shell heterostructures with different weight ratios of BaTiO_3_ toTiO_2_ demonstrated smaller surface areas via a comparison with the pure TiO_2_ nanoparticles. Among the BaTiO_3_-TiO_2_ core-shell heterostructures, the surface area decreased slightly with the increase of the weight ratio.

**Figure 2:**
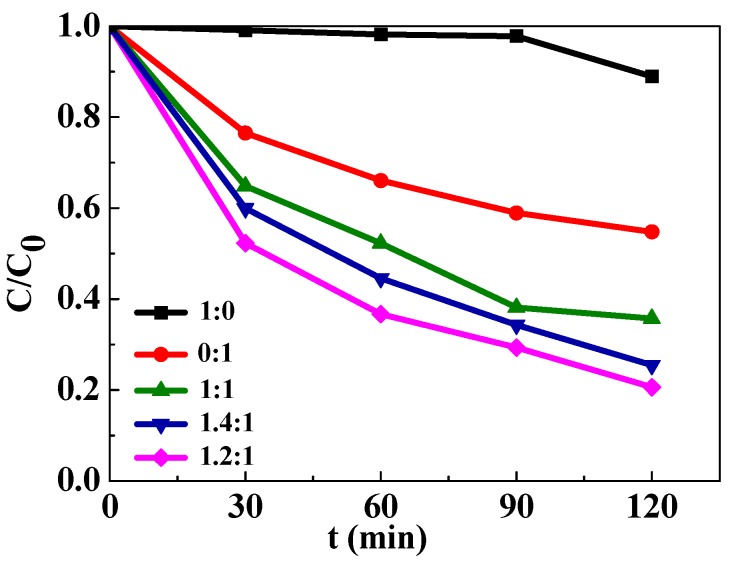
Degradation of RhB as a function of irradiation time in the presence of the as-synthesized BaTiO_3_-TiO_2_ core-shell heterostructures and TiO_2_ nanoparticles, and the commercial BaTiO_3_ crystallines under UV light irradiation. The ratios in Figure are a ratio of BaTiO_3_ to TiO_2_.

To demonstrate the effect of ferroelectric polarization on the photocatalytic activity of BaTiO_3_-TiO_2_ core-shell heterostructures, the degradation capability on RhB was evaluated under the UV light irradiation. For comparison, the photodegradation ability of the pure TiO_2_ nanoparticles and BaTiO_3_ crystallines was also evaluated at the same experimental conditions. As seen in Figure 2, the results show that slight photodegradation of RhB was detected using the pure BaTiO_3_ crystallines as the catalyst under UV light irradiation. However, all the BaTiO_3_-TiO_2_ core-shell heterostructures exhibited enhanced photocatalytic activity despite a decrease in their surface areas in comparison with the pure TiO_2_ nanoparticles. The BaTiO_3_-TiO_2_ core-shell heterostructures (1.2:1) had the best photodegradation activity, which was 1.8 times stronger than that of the pure TiO_2_ nanoparticles after a 120 min photocatalysis. The results illustrate that a BaTiO_3_-TiO_2_ heterostructure is necessary for inducing an efficient photocatalytic activity. Moreover, the weight ratio of BaTiO_3_ to TiO_2_ in the heterostructures also plays an important role in photocatalytic activity. The room temperature band gap of both BaTiO_3_ [28] and anatase TiO_2_ [29] can be taken to be 3.2 eV on the basis of the available data; therefore, difference in the light absorption of these two materials is negligible. In addition, the large decrement in *C*/*C*_0_ of the pure BaTiO_3_ crystallines at 120 min might have been caused by pyroelectric effect of BaTiO_3_ [30] because the temperature changed noticeably after 90 min irradiation of the UV light.

**Figure 3:**
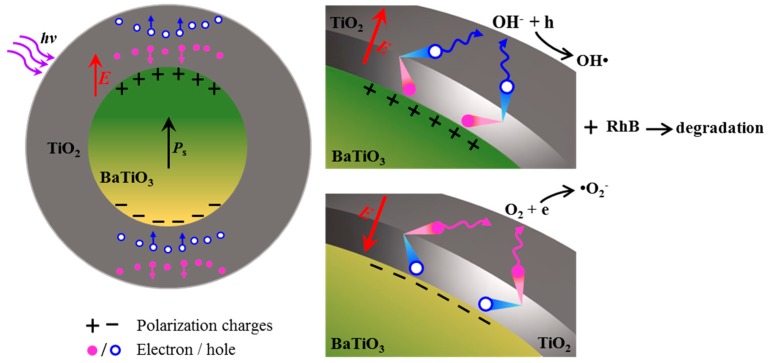
The schematic illustration of charge separation and transport facilitated by polarization-induced internal field and photodegradation of RhB based on the BaTiO_3_-TiO_2_ core-shell heterostructures.

Suppressing charge recombination and improving carrier transport are key challenges for the enhancement of the photocatalytic activity of heterostructured photocatalysts. Mesoporous TiO_2_ nanoparticles with a large surface area can benefit from the creation of numerous active sites for photocatalytic reactions; however, this process can also cause charge recombination by decreasing electron diffusion length and impeding charge transport due to the highly random surfaces/boundaries and the absence of interfacial space charges. Our concept for this work was to use ferroelectric polarization-induced internal field from the BaTiO_3_ core to facilitate charge carrier separation and further promote charge transport to the surface of TiO_2_ nanoparticles for an efficient photocatalytic reaction. Figure 3 is the schematic illustration of photogenerated charge separation and transport facilitated by polarization-induced internal field and degradation of RhB based on the BaTiO_3_-TiO_2_ core-shell heterostructures. Polarization charges in the BaTiO_3_ core can attract charges with opposite signs in the TiO_2_, which move toward the core; meanwhile, the same sign charges are repelled to the outer shell. An internal electric field can be created in the heterostructure because the polarization charges with a high density in the BaTiO_3_ cannot be fully compensated by low-concentration free charges in the adjacent TiO_2_. The average internal electric field formed from the BaTiO_3_ crystallines can be estimated by adopting the classical dipole field model [31]

(1) $$E=\;\frac{4\pi }{\varepsilon }\sigma \;f=\;\frac{4\pi }{\varepsilon }\;P_{s\;}n\;f$$

where *ε* is the relative permittivity of TiO_2_, *σ* is the polarization charge density, *n* is the unit normal to the surface, and *f* is the volume fraction occupied by the BaTiO_3_ nanocrystallines. Taking *ε* = 100 and *f* = 1 yield an estimated electric field of ~40 V/µm. This polarization-induced internal field generated locally in the heterostructure can penetrate the mesoporous TiO_2_ with a thickness less than the depletion width; it can also act as a driving force for separating electron-hole pairs and promoting them to transport toward to degradation reaction on the TiO_2_ surfaces [32]. As a result, the BaTiO_3_-TiO_2_ core-shell heterostructures with smaller surface areas present enhanced photodegradation activities compared with the pure TiO_2_ nanoparticles used as the catalyst. The dependence of photocatalytic activities on weight ratio might be relevant to the configuration of the core-shell structures. Too few or too many of BaTiO_3_ can lead to inharmonious configurations with the formation of either thicker TiO_2_ shells (>100 nm) or BaTiO_3_ aggregates with multiple crystallines, thus weakening the effect of polarization-induced internal field from the BaTiO_3_ core.

**Figure 4:**
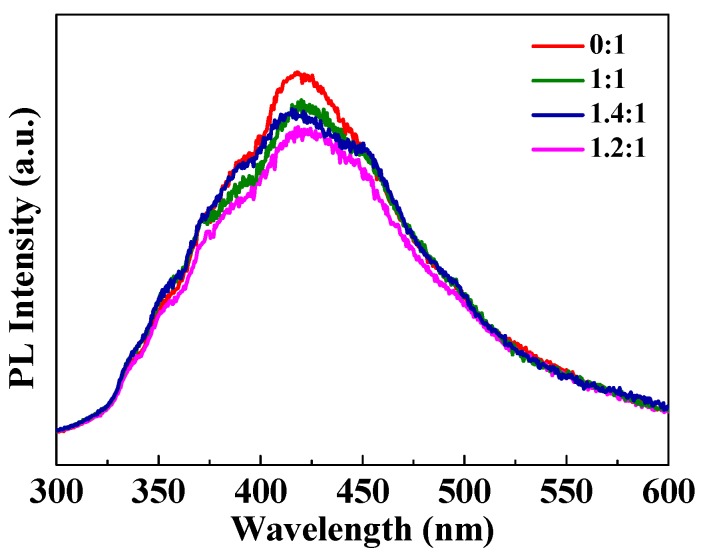
Photoluminescence spectra of the pure TiO_2_ nanoparticles and the BaTiO_3_-TiO_2_ core-shell heterostructures excited at 325 nm. The ratios in Figure are the ratio of BaTiO_3_ to TiO_2_.

PL signals of semiconductor materials result from the recombination of photogenerated charge carriers. In general, the lower the PL intensity, the lower the recombination rate of photogenerated electron-hole pairs and the higher the photocatalytic activity of semiconductor photocatalysts. In this work, PL spectra are employed to monitor the influence of ferroelectric polarization-induced internal field on the dynamic behaviors of photogenerated charge carriers in the BaTiO_3_-TiO_2_ core-shell heterostructures. As shown in Figure 4, PL intensities of all the BaTiO_3_-TiO_2_ core-shell heterostructures decreased in comparison with those of the pure TiO_2_ nanoparticles, which confirms the decrease in recombination rate of photogenerated charge carriers in the presence of light illumination. Among the heterostructures, the weight ratio of 1.2:1 exhibits the lowest PL intensity due to the lower recombination rate of photogenerated electron-hole pairs than the others. This explains the improved photodegradation activity, which is in agreement with the photocatalysis characteristics, as shown in Figure 2.

**Figure 5:**
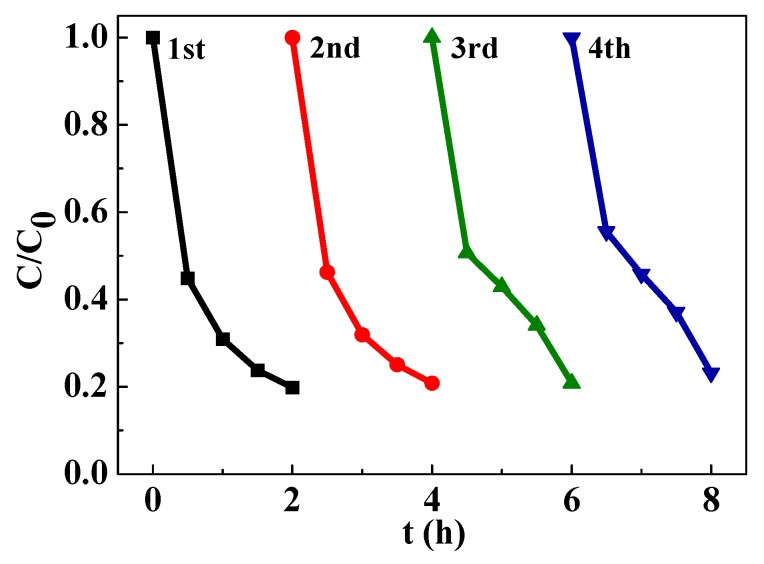
The cyclic degradation curves of the BaTiO_3_-TiO_2_ core-shell heterostructures. The BaTiO_3_ to TiO_2_ ratio is 1.2:1.

The BaTiO_3_-TiO_2_ core-shell heterostructures (1.2:1) that has the best photodegradation activity was used to evaluate the stability of photcatalysis by checking their cyclic degradation ability. As shown in Figure 5, a small reduction (~3%) in the photodegradation activity was detected after four consecutive cycles of photocatalysis. The results demonstrate that the BaTiO_3_-TiO_2_ core-shell heterostructures possess good stability of photogenerated charge separation and transport during long-term operation which can be attributed to the polarization-induced internal field.

## 4. Conclusions

In summary, we fabricated BaTiO_3_-TiO_2_ core-shell heterostructures with a varied weight ratio of BaTiO_3_ to TiO_2_ for photodegradation of RhB. The mesoporous TiO_2_ nanoshells were formed on the BaTiO_3_ core by optimizing the hydrolysis reaction followed by the hydrothermal process. The BaTiO_3_-TiO_2_ core-shell heterostructures exhibited considerably higher photocatalytic activity than the pure TiO_2_ nanoparticles, with a maximum 1.8 times enhancement obtained from the heterostructures with the optimal weight ratio of 1.2:1. Such an enhanced photocatalytic activity is attributed to the facilitation of charge separation and transport based on the internal electric field originated from the spontaneous polarization of ferroelectric BaTiO_3._ No remarkable reduction of photocatalytic activity was observed after four consecutive cycles, indicating good stability of polarization-enhanced photocatalysis for the BaTiO_3_-TiO_2_ core-shell heterostructures. This research demonstrated that the ferroelectric polarization could be an effective approach to enhance performance of heterostructured photocatalysts.

## Figures and Tables

**Table 1:** Surface areas of the samples.

Catalysts	Pure BaTiO_3_	Pure TiO_2_	BaTiO_3_-TiO_2_ Core-Shell Heterostructures		
			1:1	1.2:1	1.4:1
Surface area (m^2^/g)	25.66	70.79	36.81	35.77	34.25

## Supplementary Materials

The following are available online at https://www.mdpi.com/2079-4991/9/8/1116/s1, Figure S1. Crystal structure of (a) cubic (paraelectric) and (b) tetragonal (ferroelectric) BaTiO_3_. Figure S2. FESEM image (a) and (b) representative phase-voltage hysteresis loop (red) and amplitude-voltage butterfly loop (black) of BaTiO_3_ after subjected to the hydrothermal process. Figure S3. Photodegradation of RhB in the presence of catalysts synthesized at the different amount of TIP and NH_3_·H_2_O, and the varied stirring rate during the hydrolysis reaction Figure S4. Line-scan EDS profile (b) along the red dot dash line crossing the BaTiO_3_-TiO_2_ core-shell heterostructure (a). Figure S5. Absorption spectra of RhB solution in the presence of BaTiO_3_-TiO_2_ core-shell heterostructures (1.2:1) under UV light irradiation. Inset shows color changes of the RhB solution.
