# Supplementary material for: Ferroelectric Polarization-Enhanced Photocatalysis in BaTiO3-TiO2 Core-Shell Heterostructures
Source: Nanomaterials (Basel). 2019 Aug 3;9(8):1116. doi: 10.3390/nano9081116 (PMC6722819; doi:10.3390/nano9081116)
Supplement: Supplementary file 1 [file nanomaterials-09-01116-s001.pdf]

Article

Ferroelectric Polarization-Enhanced Photocatalysis in $BaTiO_3$-TiO_2 Core-Shell Heterostructures

Xiaoyan Liu [1,2,*], Siyi Lv [1], Baoyan Fan [1], An Xing [1] and Bi Jia [1]

[1] Chongqing Key Laboratory of Nano/Micro Composites and Devices, College of Metallurgy and Materials Engineering, Chongqing University of Science and Technology, Chongqing 401331, China

[2] Shenzhen Key Laboratory of Nanobiomechanics, Shenzhen Institutes of Advanced Technology, China Academy of Sciences, Shenzhen 518055, China

* Correspondence: xyliu@cqust.edu.cn

Received: 17 July 2019; Accepted: 30 July 2019; Published: date

Spontaneous polarization of ferroelectric $BaTiO_3$

The most extensively studied ferroelectrics are perovskite oxides (ABO_3). $BaTiO_3$ is the first identified ferroelectric material with perovskite structure. Figure S1 shows the crystal structure of $BaTiO_3$ at high temperature (> T_c, Curie point) and low temperature (< T_c). The crystal displays a cubic paraelectric phase at the temperature above T_c (120 °C). When the temperature is below T_c, Ti ions shift along the [001] axis to form a tretaganol ferroelectric phase, producing a spontaneous polarization (P_s ~27 μC/cm^2) [1].

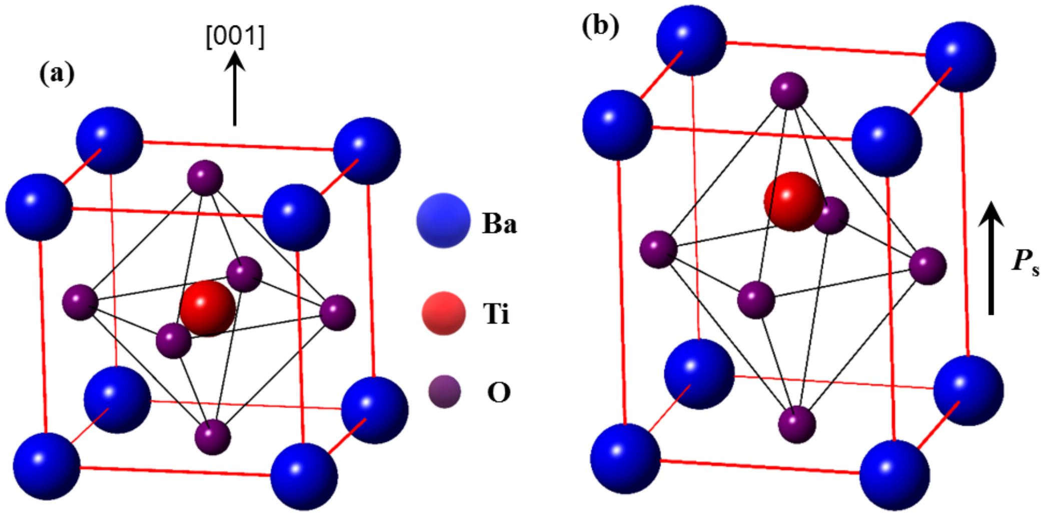

Figure S1. Crystal structure of (**a**) cubic (paraelectric) and (**b**) tetragonal (ferroelectric) $BaTiO_3$.

Characterization of ferroelectricity of $BaTiO_3$ crystallines

Commercial $BaTiO_3$ crystallines were subjected to the hydrothermal process at the absence of titanium isopropoxide (TIP) while keeping the other experimental conditions unchanged. Morphology of the $BaTiO_3$ crystallines were characterized by a field emission SEM (FESEM), showing their size range of 100 nm to 300 nm (Figure S2a). Reversible polarization and piezoelectric response of the $BaTiO_3$ were demonstrated by phase-voltage hysteresis and amplitude-voltage butterfly loops acquired from a switching spectroscopy piezoresponse force microscopy SSPFM (Figure S2b), indicating a good ferroelectric property of the $BaTiO_3$.

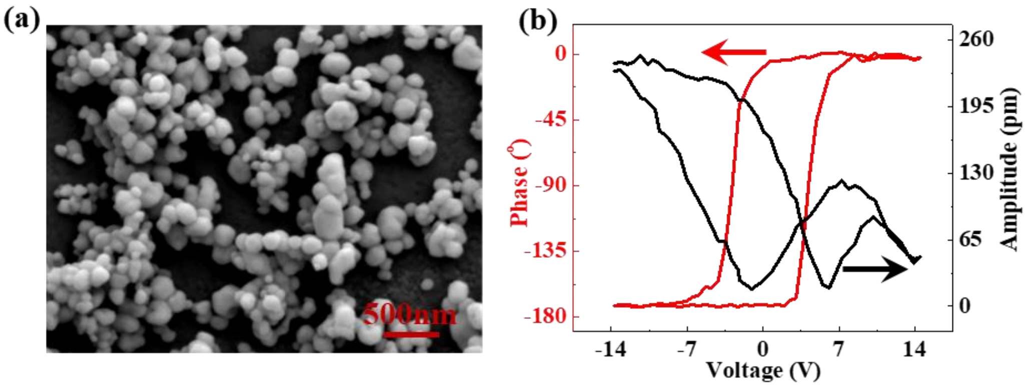

Figure S2. FESEM image (**a**) and (**b**) representative phase-voltage hysteresis loop (red) and amplitude-voltage butterfly loop (black) of $BaTiO_3$ after subjected to the hydrothermal process.

Hydrolysis reaction for forming mesoporous TiO_2 nanoshells on $BaTiO_3$

$NH_3 \cdot H_2O$ is used as a catalyst for promotion of the hydrolysis reaction of TIP, the precursor of TiO_2 [2]. The amount of TIP and $NH_3 \cdot H_2O$, and the stirring rate during the hydrolysis reaction were varied in order to coat mesoporous TiO_2 nanoshells on $BaTiO_3$. Based on the photodegradation of RhB in the presence of catalysts synthesized at the different amount of TIP and $NH_3 \cdot H_2O$, and the varied stirring rate (Figure S3), the optimal condition of the hydrolysis reaction was concluded as 0.16 ml of TIP with 0.1 ml of $NH_3 \cdot H_2O$ at the stirring rate of 200 rpm during the hydrolysis reaction.

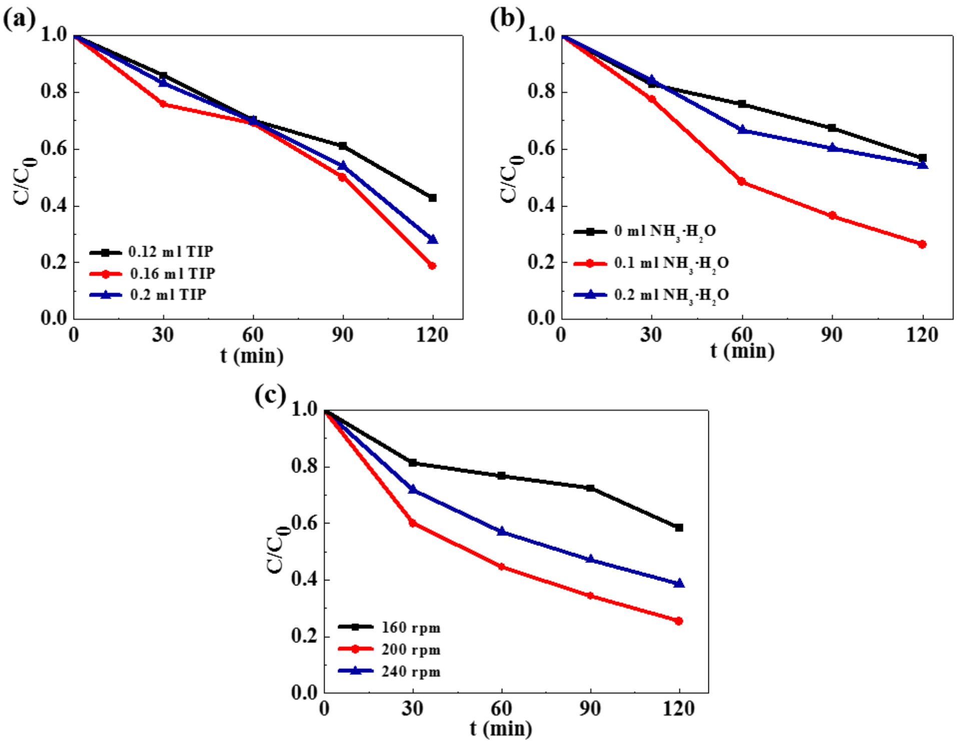

Figure S3. Photodegradation of RhB in the presence of catalysts synthesized at the different amount of TIP and $NH_3 \cdot H_2O$, and the varied stirring rate during the hydrolysis reaction.

EDS mapping of $BaTiO_3$-TiO_2 core-shell heterostructures

An energy-dispersive X-ray spectroscopy (EDS) line-scan profile across the representative individual $BaTiO_3$-TiO_2 core-shell heterostructure shows the distribution of Ba localized in the core, while the distribution of Ti and O is throughout the structure. In combination with the FESEM image (Figure S4a), the line-scan EDS profile (Figure S4b) indicates that the heterostructure consists of $BaTiO_3$ core surrounded by TiO_2 shell.

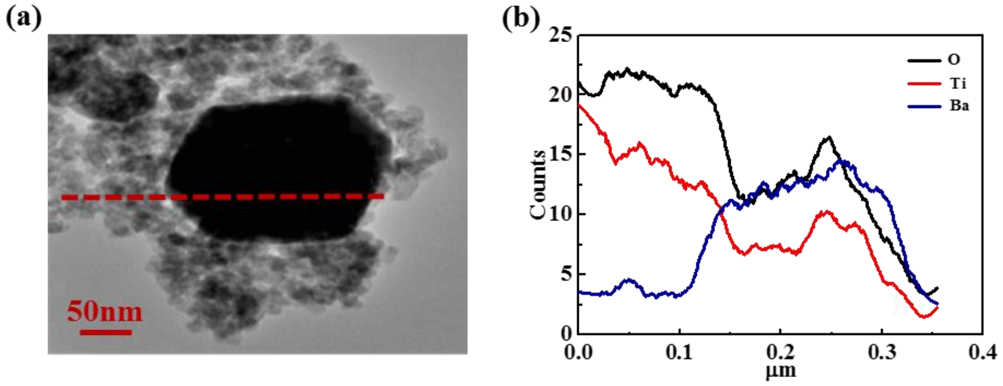

Figure S4. Line-scan EDS profile (b) along the red dot dash line crossing the $BaTiO_3$-TiO_2 core-shell heterostructure (a).

Absorption spectra of RhB solution after photodegradation

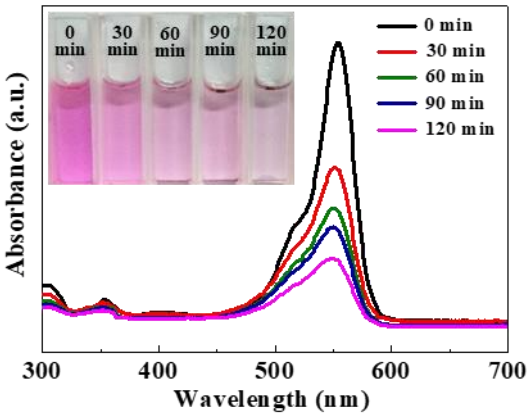

Figure S5. Absorption spectra of RhB solution in the presence of $BaTiO_3$-TiO_2 core-shell heterostructures (1.2:1) under UV light irradiation. Inset shows color changes of the RhB solution.

Photodegradation of RhB based on $BaTiO_3$-TiO_2 core-shell heterostructures

Electron-hole pairs were photogenerated mostly from the TiO_2 shell under the UV light irradiation. An internal electric field originated from spontaneous polarization of the $BaTiO_3$ core can penetrate through the TiO_2 shell with a thickness less than 100 nm and work as a driving force for separating photogenerated electron-hole pairs and promoting carrier transport to the surface of TiO_2 [3].

During the photocatalytic process, photogenerated electrons (e^-) on the surface of TiO_2

attract oxygen molecules in RhB solution and produce superoxide radicals of $\bullet O_2^-$ as shown in the following equation

$$O_2 + e^- \rightarrow \bullet O_2^- \quad (1)$$

On the other hand, photogenerated holes (h^+) react with OH^- in the solution to form hydroxyl radicals of OH•

$$OH^- + h^+ \rightarrow OH\bullet \quad (2)$$

The degradation of RhB is caused by the superoxide radicals ($\bullet O_2^-$) and hydroxyl radicals (OH•) through electrochemical redox reaction in RhB solution governed by the following relation

$$\bullet O_2^-/OH\bullet + RhB \rightarrow degradation \quad (3)$$

The reactions described above repeat over the time during the photocatalytic process.

Supplementary References

1. Husimi, K. Ultra-Low-Velocity Component of Spontaneous Polarization in BaTiO3 Single Crystal. *Journal of Applied Physics* **1958**, *29*, 1379-1380, doi:10.1063/1.1723453.
2. Guan, B.Y.; Yu, L.; Li, J.; Lou, X.W. A universal cooperative assembly-directed method for coating of mesoporous TiO₂ nanoshells with enhanced lithium storage properties. *Science Advances* **2016**, *2*, e1501554, doi:10.1126/sciadv.1501554.
3. Burbure, N.V.; Salvador, P.A.; Rohrer, G.S. Photochemical Reactivity of Titania Films on BaTiO3Substrates: Origin of Spatial Selectivity. *Chemistry of Materials* **2010**, *22*, 5823-5830, doi:10.1021/cm1018025.
